# The Baltimore College of Dental Surgery

**Published:** 1897-04

**Authors:** 


### THE BALTIMORE COLLEGE OF DENTAL SURGERY.

The 57th annual commencement exercises of the oldest school of dentistry in the world—the Baltimore College of Dental Surgery—were given in the main auditorium of the Academy of Music, March 19th, 1897, at 3 o'clock.

The graduates appeared in cap and gown, some wearing the Psi Psi Phi fraternity colors of lavender and green, and others merely the college colors of yellow and blue, arranged in small ribbons at their button-holes. A large frame in the lobby of the theatre inclosed portraits of all the graduates, taken in their academical costume, and pictures of several of the college professors.

When the curtain was raised, the graduates were greeted by a large audience, composed principally of the ladies of their acquaintance, and by a beautiful array of great banks of flowers lining the footlights. Music appropriate to the occasion was supplied by the orchestra, under the direction of Prof. John Itzel.

The exercises commenced with the announcement of the names of the graduates, made by Prof. M. Whildin Foster, M. D., D. D. S., dean of the college, by authority of the charter granted to the institution by the Legislature of Maryland in 1839. The graduates were 63 in number, their names being :

T. G. Ammenheuser, Maryland; W. Avery, South Carolina; E. L. Bartley, Pennsylvania; E. C. Baxter, Pennsylvania, E. F. Bergot, Pennsylvania; W. R. Bibber, Maine; H. E. Black, Canada; F. L. Bordelon, Louisiana; A. H. Bourque, Canada; A. L. Boyers, West Virginia; F. F. Brown, Texas; R. H. Brumby, Louisiana; C. H. Carson, Maryland; R. L. Carter, Missouri; J. H Collins, Georgia; C. S. Conn, Pennsylvania; V. Cochel, Maryland; W. F. Cook, New Jersey; W. A. Crane, New Jersey; F. L. Crie, Maine; G. B. Dantzler, Mississippi; W. L. DeLaperriere, Georgia; S. A. Demarest, New York; G. De Nux, Louisiana; L. M. Donaldson, Pennsylvania; J. W. Eddy, New York; F. W. Eheart, Virginia; F. J. Ernst, Pennsylvania; W. G. Fanning, Maine; J. H. Foote, Jr., New York; E. C. Gledhill, Rhode Island; C. S. Gore, Maryland; C. W. Granger, North Carolina; A. E. Guptill, Maine; N. Hoddes, Germany; W. N. Hoelzel, Ohio; W. E. Holidayoke, Ph. G., Maryland; A. P. Johnson, Montana; W. A. Kelly, Virginia; C. H. Kent, Vermont; M. D. Lafayette, New York; E. G. Lee, Virginia; M. M. Lindsay, Tennessee; C. Mankin, Tennessee; E. F. Martin, North Carolina; F. C. McLane, New Jersey; G. D. Morgan, Pennsylvania; E. L. Munroe, Florida; L. A. Myer, Maryland; R. R. Myers. Oregon; L. F. Palmer, Maryland; C. P. Pitt, Massachusetts; W. W. Price, Virginia; J. J. Shannon, Pennsylvania; C. W. Rink, Pennsylvania; F. W. Shores, Maine; G. Stampfl, Germany; G. B. Tison, Florida; H. N. Walters, North Carolina; D. F. Watson, North Carolina; J. W. Williams, Connecticut; J. G. Yingling, Ohio; S. J. Zufall, Pennsylvania.

Then followed, in the order given, a prayer by Rev. Dr. G. B. Miller, pastor of Grace M. E. Church, the conferring of degrees upon the graduates by Professor Foster, the dean, and the awarding of class honors by Prof. B. Holly Smith.

The diamond and gold medals, representing the first and second honors, and supplied by the faculty, were awarded, respectively, to A. E. Guptill, of Maine, whose average was 58½, and to W. A. Crane, of New Jersey, who reached 57¾. Honorable mention was made of the names of Messrs. A. P. Johnson, W. G. Fanning, F. W. Shores, J. H. Collins, L. M. Donaldson, G. Stampfl, F. C. McLane, J. W. Williams, G. B. Dantzler, A. H. Bourque, C. H. Carson, C. S. Conn, E. F. Bergot, W. A. Kelley and S. C. Demarest.

The special prizes, given by the S. S. White Dental Manufacturing Company for operative dentistry; the Snowden & Cowman Manufacturing Company, for mechanical dentistry, and the S. S. White Dental Manufacturing Company, for bridge-work, and the prize for orthodontia, were awarded to Messrs. G. B. Tison, A. L. Boyers, G. B. Tison and A. E. Guptill, respectively. Very honorable mention was given Mr. F. F. Brown in operative dentistry, Mr. F. W. Shores in mechanical dentistry, and F. L. Bordelon in bridgework, and honorable mention to Mr. L. F. Palmer in operative dentistry, and Mr. C. S. Gore in orthodontia.

The exercise concluded with the annual oration by Rev. Dr. D. B. Greigg, pastor of the Twelfth Presbyterian Church of Baltimore, who gave the newly fledged dentists much good advice, and the valedictory by Dr. Frederick C. McLane, of New Jersey.

The graduating class officers are: W. Alanson Crane, of New Jersey, president; Emil F. Bergot, of Pennsylvania, secretary; Gordon B. Tison, of Florida, treasurer; Frederick C. McLane, of New Jersey, valedictorian, and Alphonso H. Bourque, of Canada, class artist. Messrs. Edwin G. Lee, of Virginia, chairman; R. Hainsworth Brumby, of Louisiana; Edward L. Munroe, of Florida; Lewis M. Dona dson, of Pennsylvania, and Forrest L. Bordelon, of Louisiana, compose the executive committee of the class.

